# Targeting p53 misfolding conundrum by stabilizing agents and their analogs in breast cancer therapy: a comprehensive computational analysis

**DOI:** 10.3389/fphar.2023.1333447

**Published:** 2024-01-10

**Authors:** Burhan Ul Haq, Hina Qayoom, Shazia Sofi, Nusrat Jan, Aisha Shabir, Irshad Ahmad, Fuzail Ahmad, Abdullah Almilaibary, Manzoor A. Mir

**Affiliations:** ^1^ Department of Bioresources, School of Biological Sciences, University of Kashmir, Srinagar, India; ^2^ Department of Medical Rehabilitation Sciences, College of Applied Medical Sciences, King Khalid University, Abha, Saudi Arabia; ^3^ Respiratory Care Department, College of Applied Sciences, Almaarefa University, Diriya, Saudi Arabia; ^4^ Department of Family and Community Medicine, Faculty of Medicine, Al Baha University, Albaha, Saudi Arabia

**Keywords:** breast cancer, mutation, p53 misfolding, aggregation, PhiKan-083, molecular docking

## Abstract

Cancer continues to be a major global public health concern and one of the foremost causes of death. Delays in the diagnosis and cure may cause an increase in advanced stage disease and mortality. The most common cancer found in women currently is breast carcinoma. Breast carcinoma has surpassed lung carcinoma and currently represents the chief type of cancer diagnosed (2.3 million new cases, which amount to 11.7% of all cancer cases). In addition, by 2040, the incidence will increase by more than 46% as per the estimates of GLOBOCAN. Triple-negative breast cancer (TNBC) represents a highly aggressive and invasive subtype of breast cancer, characterized by rapid progression, short response time to the available treatment, and poor clinical results. Thus, it is very crucial to develop novel diagnostic tools and therapeutics with good efficacy. A majority of cancers display malfunction along the p53 pathway. Moreover, p53 not only loses its function but is also prone to misfolding and aggregation, leading to formation of amyloid aggregates as well. Research is being carried out to find ways to restore the normal action and expression of p53. Here, we have explored PhiKan-083 for its possible stabilizing effect on p53 in order to address the problem with its misfolding. Thus, examining the analogs of PhiKan-083 that have a role in p53 stability will help update our understanding of cancer progression and may expedite the progress of new anticancer treatments. We anticipate that the drug molecules and their analogs targeting p53 aggregation may be used in combination with other anticancer compounds to solve the problem with p53 aggregation. In this study, by employing ADMET analysis, the compounds were screened, and we further examined the chosen compounds with the help of molecular docking. By using databases like UALCAN, TIMER, GEPIA, and PredictProtein, we investigated TP53’s expression pattern and prognostic relevance in various cancer settings.

## Graphical Abstract

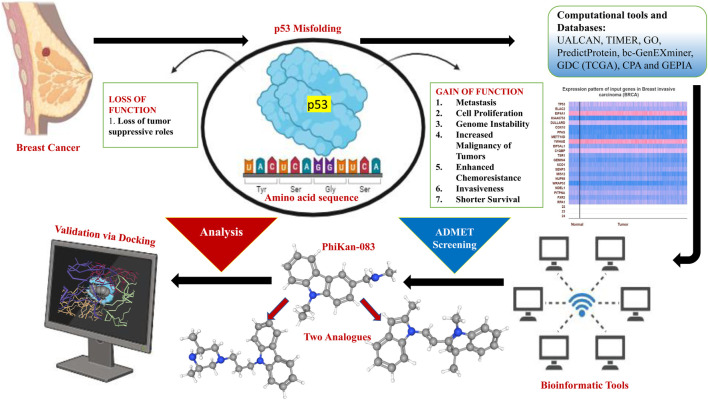

## Highlights

⁃ Breast carcinoma is one of the most frequent malignancies diagnosed globally and the chief cause of mortality among women.
⁃ Although there have been significant advances in the strategies for curing breast cancer, the poor prognosis is still a matter of concern.
⁃ Due to its heterogeneous nature, it becomes hard to access the surviving individuals.
⁃ The altered p53 protein is susceptible to misfolding and accumulation, ultimately leading to formation of amyloid aggregates.
⁃ Thus, reverting or restoring the normal p53 function is a marked strategy for possible cancer cure.
⁃ In this study, we examined PhiKan-083 and its analogs for restoring or reverting the misfolding of p53 protein. In addition, we also explored the expression pattern of misfolded p53 protein across several carcinomas, especially breast cancer.

## Introduction

Breast carcinoma (BC) is one of the most frequent malignancies diagnosed globally and the chief cause of mortality among women (Siegel et al., 2021; Mir and Qayoom, 2023). Nearly 30% of all cancer incidences in women are due to breast cancer (Sung et al., 2021). Globally, BC represents recurrent malignancy among women. In 2020, it exceeded lung cancer to become the top reason of cancer incidence worldwide. A total of 2.3 million additional cases were anticipated, accounting for approximately 11.7% of all cancer cases (Hall et al., 1990). Epidemiological studies have revealed that the burden of breast cancer globally is thought to cross nearly 2 million by the year 2030 (Wooster et al., 1994). The primary major gene that was identified to be associated with hereditary BC in 1990 is BRCA1. Additionally, it was identified by employing linkage analysis in families by exploring suggestive pedigrees (Gallanis et al., 2021). Following this, BRCA2 was mapped to chromosome 13 in 1994 (Baldin et al., 1993). An alteration in either BRCA1 or BRCA2 increases susceptibility to cancer risk, especially BC. Metastatic breast cancer remains almost completely incurable, with an average overall survival (OS) of 3 years and a merely 25% 5-year OS (Cardoso et al., 2018; Sofi et al., 2022a).

Although there have been significant advances in the strategies for curing breast cancer, the poor prognosis is still a matter of concern (Waks and Winer, 2019; Sofi et al., 2022b; Mir and Haq, 2023a; Qayoom et al., 2023a; Mir and Haq, 2023b). Due to its heterogeneous nature, it becomes hard to access the surviving individuals (Turashvili and Brogi, 2017; Mehraj et al., 2021; Qayoom et al., 2021). Multiple studies on breast cancer previously identified several genes which possess prognostic value. However, the developing field of bioinformatics along with advancing algorithms has provided researchers with the environment to discover potential genes by employing a different approach (Mehraj et al., 2022; Qayoom et al., 2023b; Sofi et al., 2023). The p53 protein has an essential role in prevention of genomic mutation in the body, but this protein is prone to mutations at a higher rate as witnessed in cancers. Moreover, a malfunction is witnessed in approximately all cancers along the p53 pathway. The altered p53 protein not only loses its function but is also susceptible to misfolding and accumulation, ultimately leading to the formation of amyloid aggregates. Protein misfolding and accumulation have a part to play in cancer development. Recognized as the “guardian of the genome,” p53 plays a crucial role in preventing genomic mutations. Structurally, p53 represents a tetrameric transcription factor that modulates multiple cellular roles like expression of target genes that lead to apoptosis, repair of DNA, and arrest of the cell cycle in damaged cells in addition to cellular metabolism (Helton and Chen, 2007; Lukin et al., 2015; Mir et al., 2023a). Due to the fact that more than 50% of cancers are caused due to direct point mutations of p53 (Petitjean et al., 2007; Olivier et al., 2010) and the p53 malfunction as seen in aggressive cancer types like gastric cancer (Blanchet et al., 2021); thus, reverting or restoring the normal p53 function is a marked strategy for possible cancer cure (Hong et al., 2014; Bykov et al., 2018; Miller et al., 2020). The levels of P53 are highly modulated through post-translational alterations and a pivotal modifier modulator mouse double minute 2 homolog (MDM2). However, during cellular stress, there is an inhibition to p53 degradation; in addition, the protein is accumulated in the nucleus (Friedman et al., 1993; Nag et al., 2013; Mir et al., 2022). The same causes target gene upregulation and triggers reaction mechanisms to bypass the multiplication of injured cells (Brooks and Gu, 2010). For example, when DNA damage occurs, p53 may either force the injured cell to be removed or trigger a cell cycle blockage mechanism that allows DNA repair to proceed (Helton and Chen, 2007; Lukin et al., 2015). The studies have revealed that the DNA-binding domain (DBD) of wild-type p53 displays less thermodynamic and kinetic stability (Natan et al., 2011) and several mutations lead to instability, ultimately causing protein unfolding. Cancer cells acquire high amounts of mutant p53 because it avoids the MDM2-mediated proteasomal destruction (Scian et al., 2004). MDM2 has been shown to play a post-ubiquitination function in the degradation of p53 (Kulikov et al., 2010). Aggregation and unfolding are intricate processes; however, destabilization exposes an adhesive sequence that attaches to the same section in other p53 units and triggers aggregation. Consequently, there is significant interest in the creation of novel agents that can both stabilize folded structures and prevent aggregation or split pre-formed aggregates (Miller et al., 2020; Miller et al., 2022). There are multiple ways through which altered p53 protein can be stabilized in its active and folded form. A comprehensive set of Michael acceptors, including CP-31398 and PRIMA1, has been seen to rectify the native information on mutant p53 and initiate p53-dependent cytotoxicity (Foster et al., 1999; Bykov et al., 2002; Rangel et al., 2019; Mir et al., 2023b). PhiKan-083 represents a small molecule and a carbazole derivative, which binds to the surface cavity and leads to the stabilization of a p53 mutant, namely, Y220C, possessing a K_d_ of 167 μM. Thus, we attempted to explore PhiKan-083 for breast cancer research. Y220C mutation, which is not involved in DNA interaction, causes a destabilization of the protein. PhiKan-083 binding stabilizes the mutant p53 and slows the rate of denaturation.

Hence, for this study, we screened and examined many analogs of PhiKan-083 with better docking scores in comparison to PhiKan-083 for stabilizing misfolded p53. We evaluated the molecule-to-molecule docking of selected compounds to determine the therapeutic potential of screened PhiKan-083-related analogs by calculating the drug-likeliness of those compounds. This work identified two novel compounds that, in addition to their potential as PhiKan-083 substitutes, have advantages such as potential activity against a variety of p53-misfolded malignancies and not just breast cancer. We also looked at p53’s expression patterns and predictive values across a range of cancer types, especially breast cancer.

## Methodology

### UALCAN

UALCAN (http://ualcan.path.uab.edu/) represents a web-based interactive portal to investigate cancer omics data (Chandrashekar et al., 2017). UALCAN has been made to furnish access to publicly available cancer OMICS data easily with the aim of running *in silico* validation of genes that are of interest or to identify related biomarkers. These databases furnish the graphical representation along with the plots displaying the expression profiles along with the subject survival information. We selected TCGA invasive breast carcinoma data, and P53 expression was examined among different cancer stages, sample types, and sub-classes.

### TIMER

TIMER 2.0 (http://timer.comp-genomics.org/) is an instructive tool used to thoroughly evaluate the expression and quantification of immune infiltrates across malignancies. Users can investigate the connection between gene expression and other variables using the database’s algorithms, including quanTIseq, CIBERSORT, MCP-counter, and EPIC algorithms; the status of its mutation; and its association with different immune cell kinds. To better comprehend the gene expression profiles of all malignancies, a heatmap of TP53 was created using TIMER 2.0 (Li et al., 2020).

### GEPIA2

Through TCGA and GTEx projects, RNA-sequencing expression data from 9,736 tumors and 8,587 normal samples were analyzed using the web-based application GEPIA2 (http://gepia2.cancer-pku.cn). Subject survival analysis, dimensionality reduction study, identification of related genes, profiling based on cancer types or pathological stages, assessment of differential expression between tumors and normal tissues, and correlation analysis are a few of the resources that can be tailored. By gathering RNA-seq datasets from the UCSC Xena project (http://xena.ucsc.edu), the GEPIA2 followed a pre-established process (Tang et al., 2019). The TP53 expression in varied cancers was obtained by employing the GEPIA2 portal.

### bc-GenExMiner

The breast cancer gene-expression miner v4.5 (http://bcgenex.ico.unicancer.fr/) is a bioinformatics tool available online that contains published BC transcriptomic and RNA-seq data in an annotated form (Jézéquel et al., 2012; Jezequel et al., 2013). It was employed to analyze the link between p53 expression levels and distinct clinical factors of breast tumor subjects. P53 expression analysis results were analyzed for basal-like luminal A and luminal B types.

### The cancer proteome atlas

For researchers who want to carefully examine the functional proteomics of various cancer types, the University of Texas MD Anderson Cancer Center’s Cancer Proteome Atlas Portal is a go-to source (https://tcpaportal.org/tcpa/). Using the website’s database of patient cohorts, the users can search through the datasets of patient tumors that have been obtained and sequenced by the Center. Users can obtain thorough explanations of each dataset and use a network model or heatmap to evaluate the interactions of the proteins in the data. This website offers a database of cancer cell lines in addition to the patient’s cohort database, which researchers can browse and analyze.

### GDC (TCGA)

The National Human Genome Research Institute (NHGRI) and the National Cancer Institute (NCI), both of which are divisions of the National Institutes of Health of the U.S. Department of Health and Human Services, have collaborated on it (https://portal.gdc.cancer.gov/). A pilot study for the Cancer Genome Atlas was conducted to see whether a larger project must be undertaken to systematically examine every type of genetic mutation connected to human cancer. Next, using matched normal samples, the scientists molecularly defined almost 20,000 initial cancer samples from 33 distinct cancer types. It is open to all members of the scientific community to use the almost 2.5 petabytes of data generated by TCGA.

### PredictProtein

Since 1992, PredictProtein (https://predictprotein.org) has predicted the structure and functional characteristics of proteins using sequence analysis. Multiple sequence alignments, anticipated structural elements [solvent accessibility, secondary structure, coiled-coil regions, transmembrane helices (TMSEG) and strands, disulfide bonds, and disordered areas], and functional elements are returned in response to a protein sequence query. Among the analysis techniques included in the service are functional region identification (ConSurf), homology-based inference of GO terms (meta-student), precise subcellular localization prediction (LocTree3), protein–polynucleotide binding sites (SomeNA), protein–protein binding sites (ISIS2), and predictions of the impact of point mutations (non-synonymous SNPs) on protein function (SNAP2). Their objective has consistently been to provide a tailored solution that satisfies the needs of researchers with less experience in bioinformatics. To achieve this, the PredictProtein results are given as texts along with a number of coherent, engaging, and visually appealing images. The sources and web server may be accessed at http://ppopen.rostlab.org.

### Screening of compounds related to PhiKan-083

In this work, PhiKan-083 served as the reference compound, and 100 compounds with similar structural properties were examined. We collected these compounds from the PubChem database (https://pubchem.ncbi.nlm.nih.gov/) and investigated all of the physico-chemical characteristics of the compounds. The specific compounds were analyzed using Discovery Studio v18 after copying them in SDF format (Kim et al., 2019).

## ADMET examination

### Drug-like dataset development

The retrieved PhiKan-083 analogs were assessed for drug-likeness using SwissADME, an online portal that assesses a drug’s potential by examining characteristics such as absorption, distribution, metabolism, excretion, and toxicity (ADMET) of the selected 100 analogs (http://www.swissadme.ch). Numerous parameters, such as hydrogen-bond acceptors (nHBAs), the log of the n-octanol/water partition coefficient (LogP), hydrogen bond donors (nHBDs), molecular polar surface area (PSA), molecular weight (MW), and number of rotatable bonds (nRotBs), were calculated by incorporating Lipinski’s Rule of 5 and the Veber rule. The ADMET prediction technique took into account a number of parameters, including aqueous solubility, cytochrome P450 (CYP) 2D6 inhibition, hepatotoxicity, intestinal absorption, and plasma protein binding. After being chosen, the compounds that complied with ADMET drug-likeliness guidelines underwent molecular docking (Rudrapal et al., 2022).

### Selection of a target and its preparation

The target protein TP53 (PDB ID 8dc4) was obtained from the Protein Data Bank (PDB) in a PDB format (Kulikov et al., 2010). By removing the extra structural moieties like heteroatoms, water molecules, and any associated ligands, the p53 protein was cleared up for further study using the Discovery studio (Nguyen et al., 2014).

### Molecular docking

To ascertain the ligands’ affinities for binding to TP53, the compounds were first exposed to molecular docking after undergoing ADMET analysis. Every ligand was docked with the TP53 protein using AutoDock v4.2.6. By using the co-crystallized X-ray structure of the p53 protein from the RCSB PDB, the binding cavity was built. The co-crystallized ligand’s three spacers’ residue locations were computed. Following the selection of a cavity, the energy was reduced by applying conjugate gradient and steepest descent algorithms. By merging the nonpolar hydrogens, the receptor and target molecules were finally stored in a pdbqt format. We then generated the grid boxes at 0.3 intervals. The protein–ligand complex was docked using the Lamarckian genetic algorithm (LGA) in order to determine the lowest free energy of binding (G). Three similar sets of molecular modeling experiments were carried out, with the default settings being a maximum of 2,700 generations, 2,500,000 evaluations, and 50 solutions each set (Qayoom et al., 2022).

## Results

### TP53 is highly upregulated across pan-cancer

**FIGURE 1:**
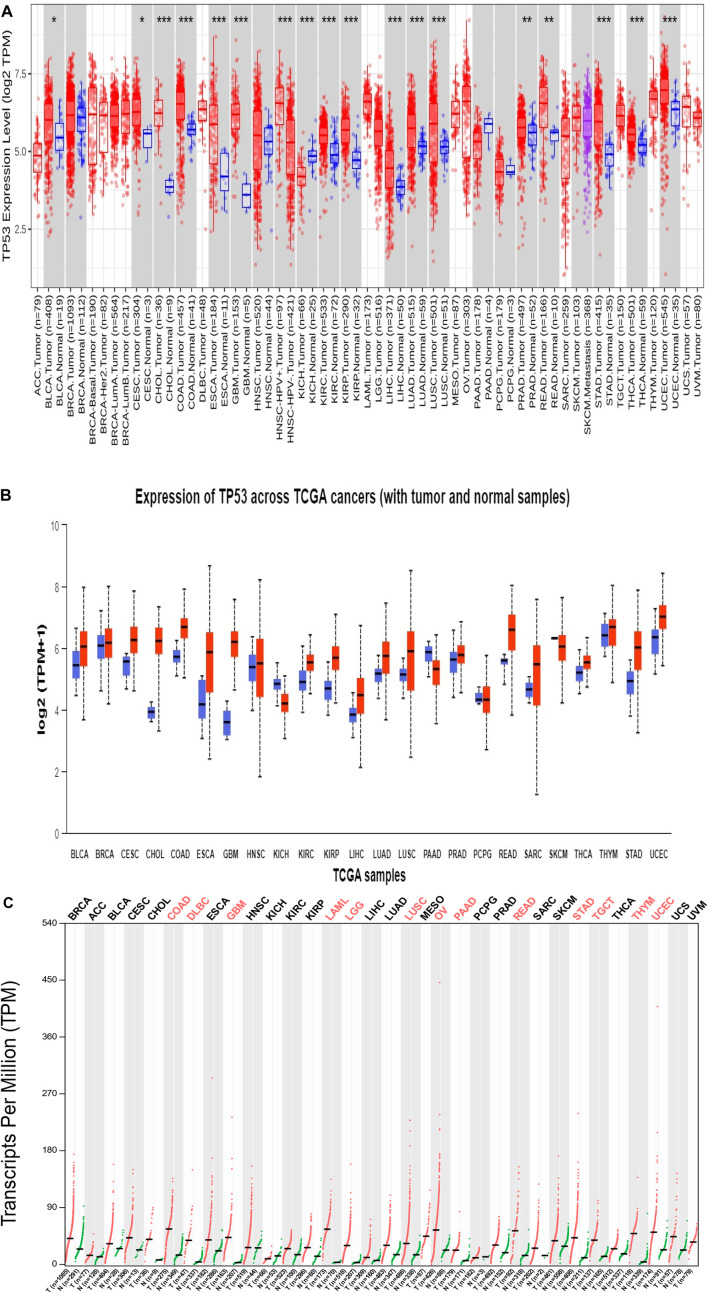
(Continued).

**FIGURE 2:**
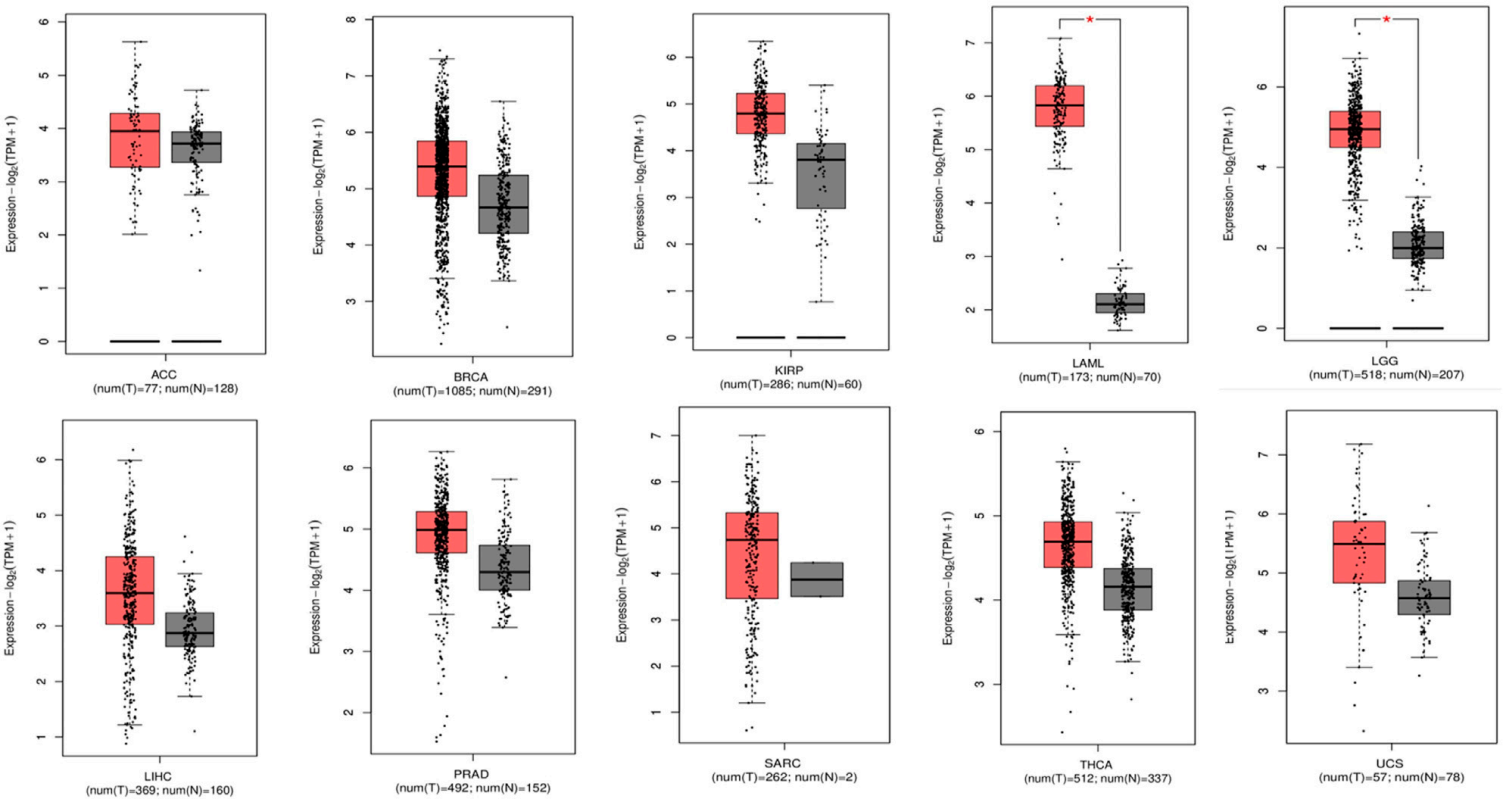
Box plots representing individual carcinomas, each having overexpressed P53 levels revealing the significance of P53 as an important target (gray color: normal sample; red color: tumor sample).

As shown in the heatmap in Figure 1A, the TIMER 2.0 analysis of the TP53 expression pattern showed overexpression of TP53 across all TCGA datasets. TP53 levels were further assessed using CPTAC samples from the UALCAN database. When compared to normal samples, it was also discovered that the levels of the protein TP53 were significantly increased in a number of tumor samples Figure 1B. The majority of malignancies showed the pattern, with elevated TP53 protein levels seen in the samples of UCEC, READ, THYM, COAD, CHOL, CESC, BRCA, and BLCA when compared to normal samples. By employing the GEPIA2 database, we also analyzed the expression pattern of TP53 in various cancers (Figure 1C). Our results revealed that TP53 is significantly increased in a number of malignancies, including BLCA, ESCA, BRCA, BRCA basal, BRCA luminal A, BRCA luminal B, and BRCA HER2. Other cancers with TP53 upregulation include TGCT, THYM, UCS, UCEC, GBM, DLBC, COAD, CHOL, CESC, OV, and STAD (Figure 2). Furthermore, it was witnessed that in contrast to other malignancies, BLCA, BRCA, BRCA basal, BRCA luminal A, BRCA luminal B, BRCA HER2, COAD, HNSC, LAML, OV, UCEC, and STAD displayed high mRNA levels of TP53.

### Gene ontology analysis

**FIGURE 3:**
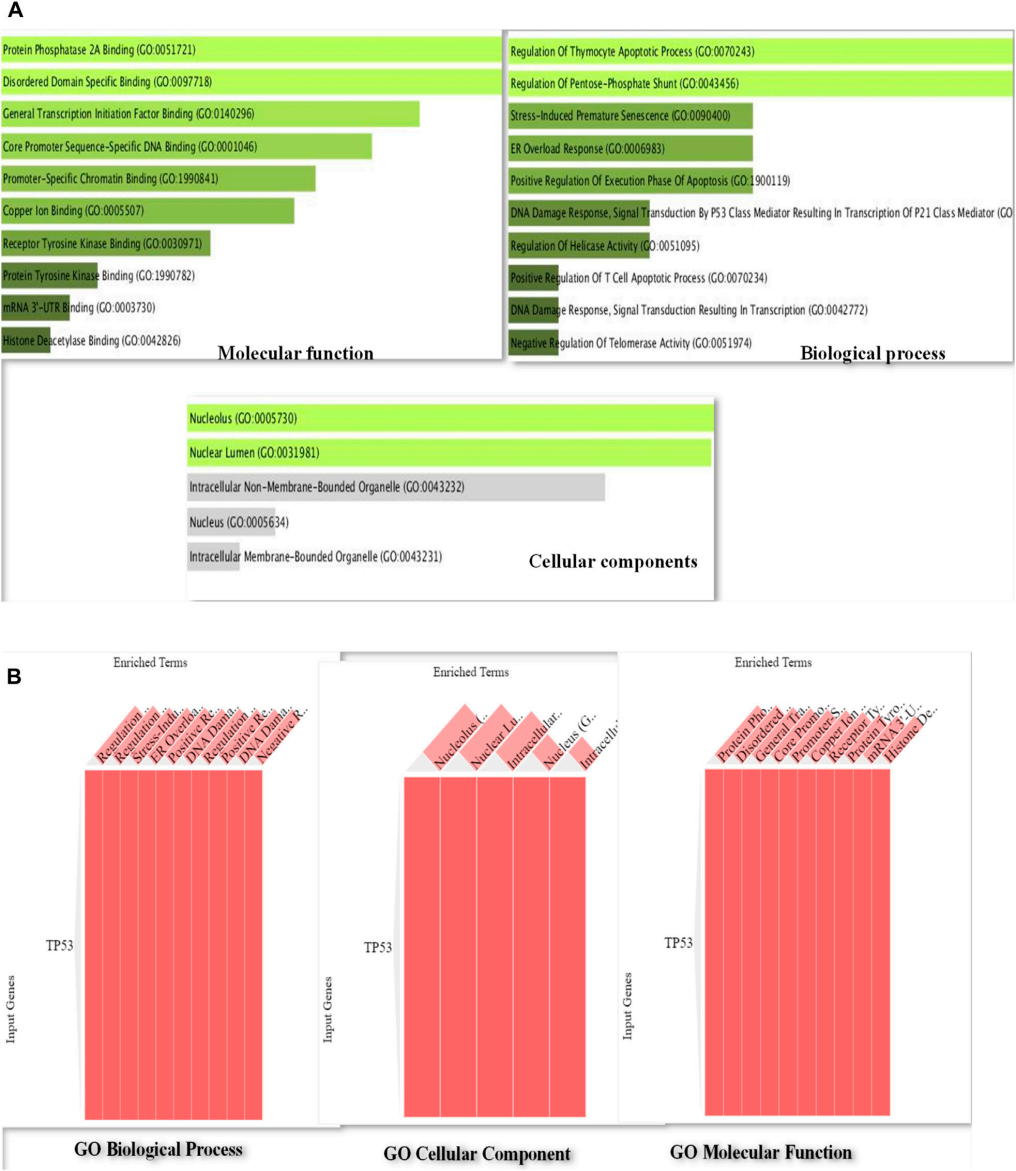
**(A)**: Bar diagram of Gene Ontology analysis of P53 protein representing the involvement of P53 in important pathways such as regulation of apoptosis execution phase, DNA damage response, and signal transduction and helicase activity. **(B)**: Gene Ontology cluster diagram representing the extent of the impact of P53 on several important pathways including molecular, cellular, and biological processes.

By using the web-based Enrichr program for GO analysis, it was demonstrated that MF was considerably enriched compared to BP and CC. Figure 3 illustrates the various processes in which TP53 was found to be actively involved among the MF, including binding of protein phosphatase 2A, specific binding to disordered domains, binding of general transcription initiation factors, DNA binding specific to core promoter sequences, and promoter-specific binding. TP53 was implicated in several biological processes, including thymocyte apoptotic process regulation, pentose–phosphate shunt regulation, stress-induced premature senescence, ER overload response, positive regulation of apoptosis execution phase, DNA damage response, signal transduction, P53 class mediator leading to P21 class mediator transcription, and helicase activity figure regulation. Figures 3A, B show that TP53 was abundant in the nucleolus and nuclear lumen in the CC terms of the GO analysis.

### Prognostic importance of TP53 across pan-cancers

**FIGURE 4:**
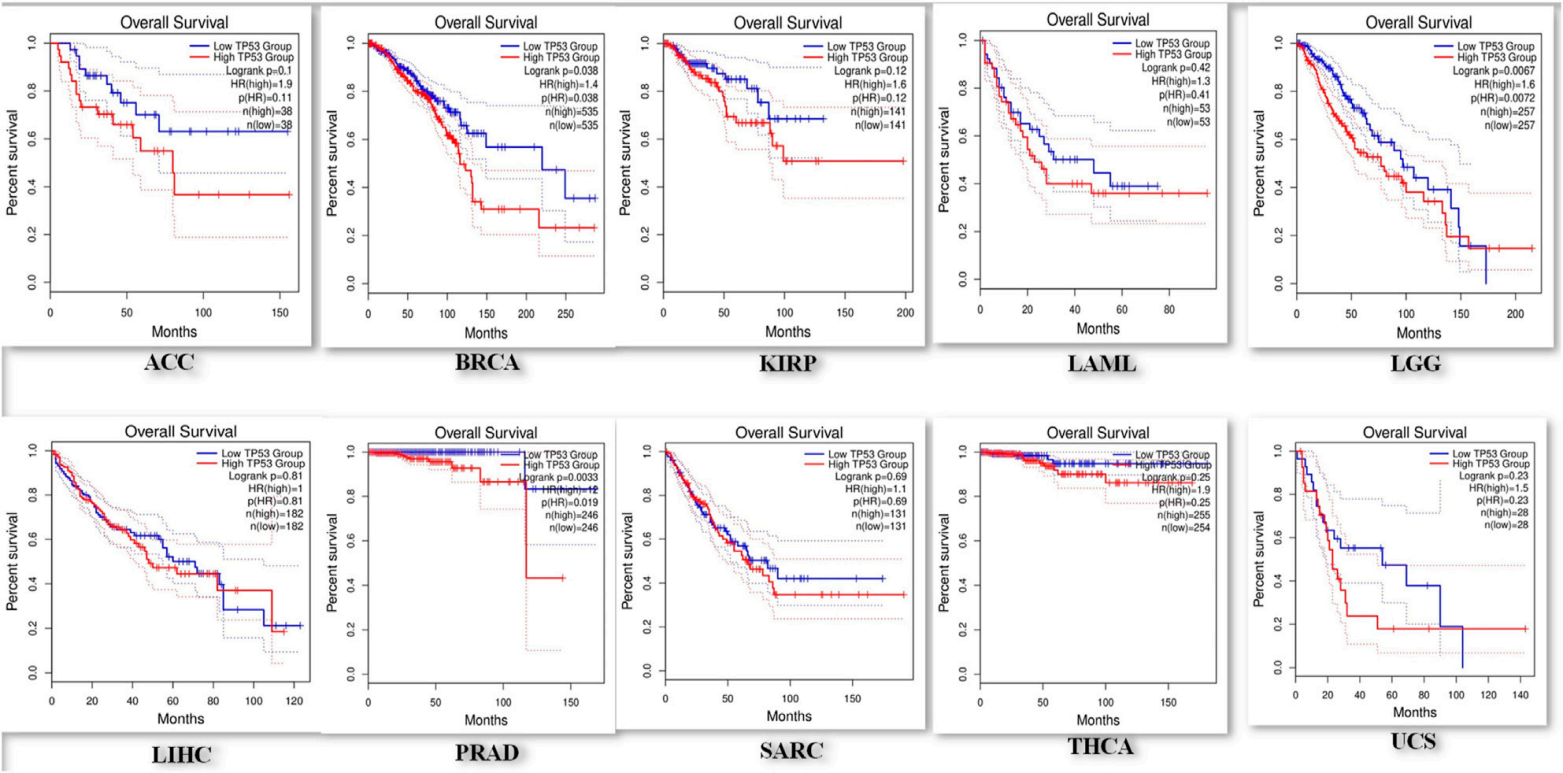
Representation of a decreased overall survival of pan-cancer patients with increased overexpression in P53 levels.

Using the KM-plotter from GEPIA2, the prognostic significance of TP53 was assessed across all cancer types. The relationship between OS and relapse-free survival (RFS) and the median expression of TP53 was investigated. According to the study, reduced OS is associated with elevated TP53 expression in ACC, BRCA, KIRP, LAML, LGG, LIHC, PRAD, SARC, THCA, and UCS (Figure 4). The hazard ratio for all malignancies ranged from 1 to 12, with LIHC having the lowest and PRAD having the highest. High TP53 mRNA levels are linked to worse RFS in LGG, UCS, and BRCA, followed by LAML, SARC, ACC, KIRP, LIHC, PRAD, and THCA according to the connection between TP53 mRNA levels and RFS. These investigations demonstrated the importance of TP53 in cancer prognosis, making it a promising target for treating various malignancies.

### Analysis of p53 expression in BC

**FIGURE 5:**
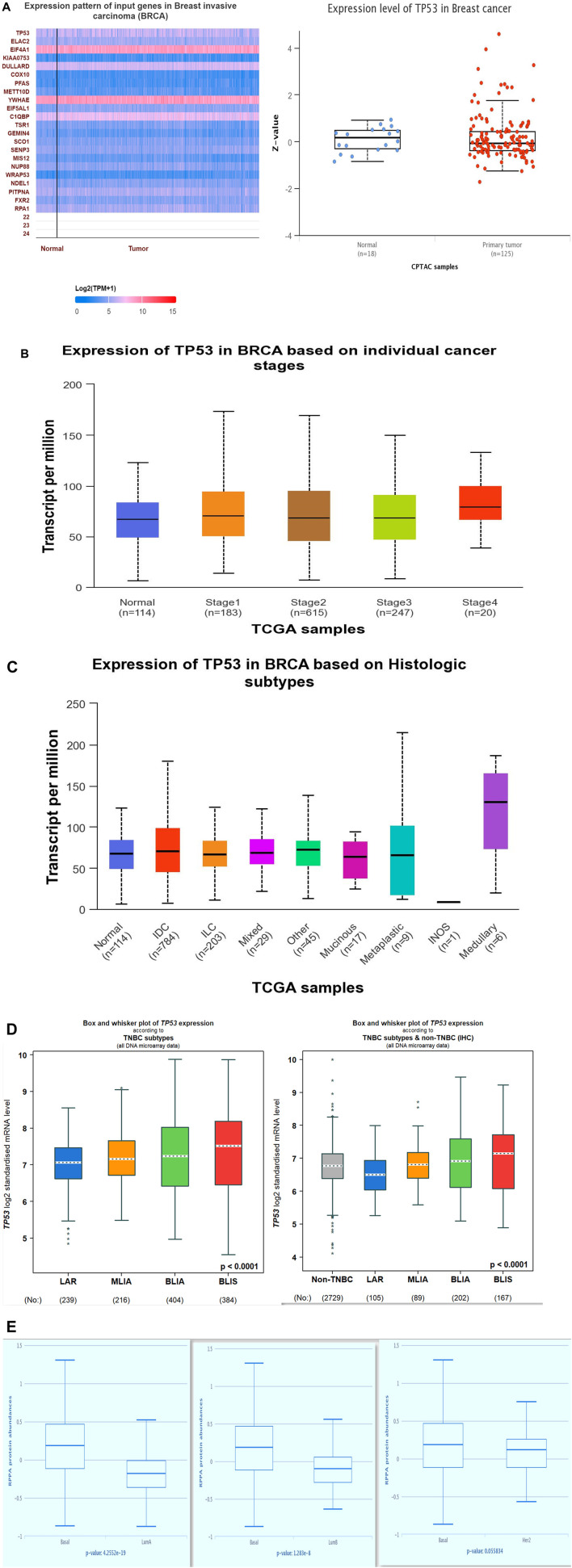
(Continued).

Using the UALCAN database, we analyzed the expression pattern of p53 in breast cancer. The heatmap was generated by correlating p53 with other overexpressed genes in BC using Pearson’s correlation coefficient (Figure 5A). The study showed that among all the upregulated genes, p53 was relatively upregulated. We compared the p53 expression in both normal and tumor samples, and p53 was found to be overexpressed in breast cancer. Using the same database, we also analyzed the expression of p53 among various stages of BC (Figure 5B), histological subtypes (Figure 5C), and breast cancer subtypes (Figures 5D, E). Since TNBC is the most aggressive subtype of breast cancer, our study revealed that among all subtypes, P53 was overexpressed in TNBC, and within TNBC, it was most prominent in the BLIS subtype of TNBC. As has been shown in another result obtained from The Cancer Proteome Atlas (TCPA), the increased expression of P53 was seen to be TNBC (basal-like) in comparison to luminal A and luminal B subtypes of breast cancer. These results validated the significance of the effects of TP53 on the aggressive nature of breast cancer, particularly triple-negative breast cancer.

### Analysis of P53 misfolding and functional status

**FIGURE 6:**
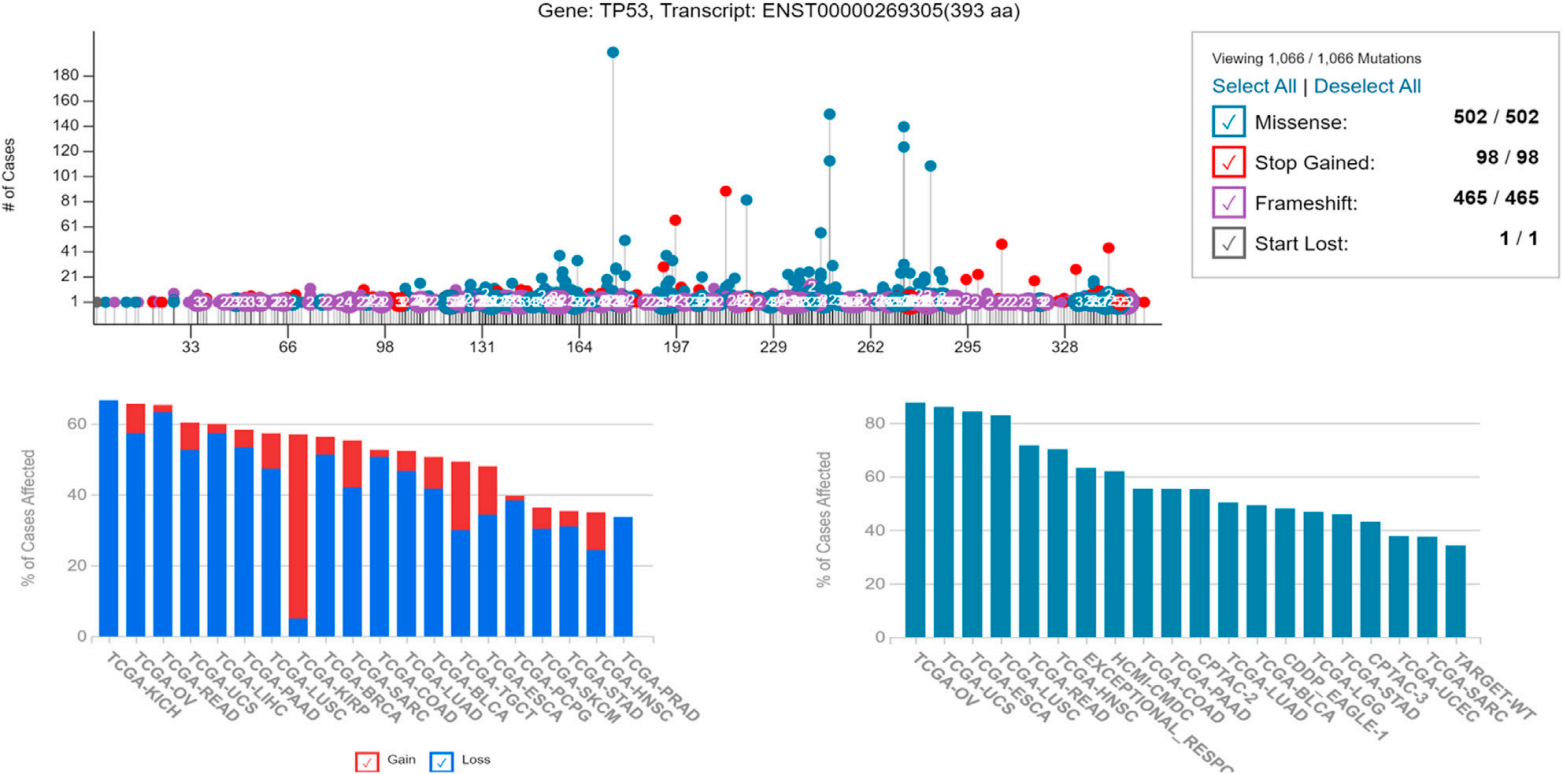
Showing the alteration in the genetic frequency of P53 revealing its gain-of-function potential, responsible for its increased tumorigenicity potential and higher percentage of affected cases due to the same.

Since the major problem associated with p53 misfolding is due to its point mutation status, therefore we analyzed the amino acid sequence of P53 using the GDC (TCGA) database to check the frequency of the mutational status of P53. It was revealed that the p53 protein sequence has a higher frequency of structural mutations that have significantly impacted its folding ability, thereby affecting its tumor suppressor function. Since protein misfolding is underplayed, it was further assessed to analyze the frequency of mutations that are non-functional or mutations that lead to gain of tumor function. An enhanced gain-of-function potential was attained in p53 protein due to its misfolding, as shown in Figure 6.

### Analysis of protein folding and stability pattern of p53 protein

**FIGURE 7:**
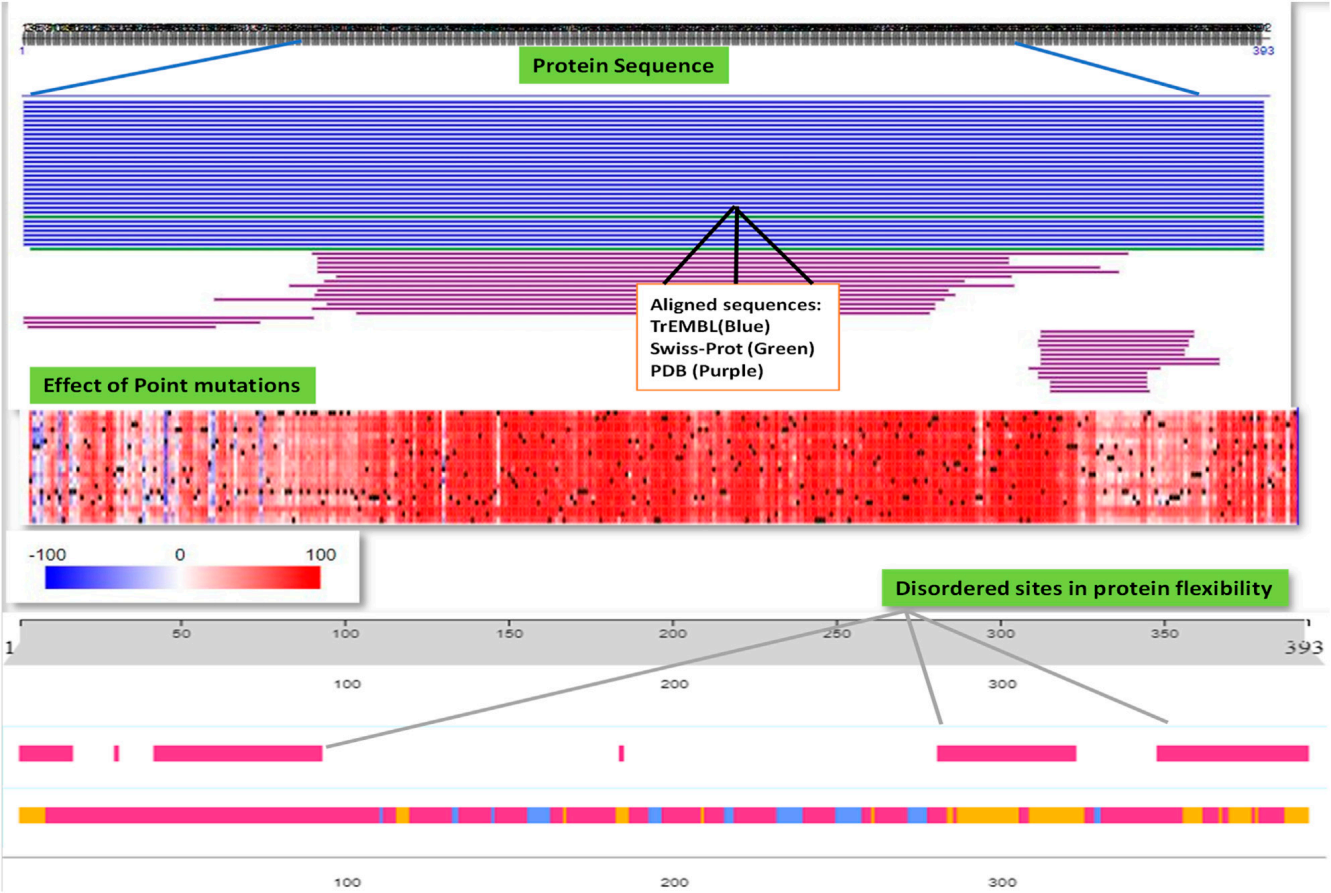
PredictProtein database was used to employ and analyze the general protein sequence of P53 and the effect of point mutations responsible for the misfolding of P53 protein possessing disordered sites aiding to its aggressiveness.

It was revealed from the above results how P53 protein expression was highly upregulated in several cancers including breast cancer, importantly TNBC, and the misfolding was directly correlated to its gain of function toward tumor development. We further explored the protein secondary structure using the PredictProtein tool and analyzed the functional effect of point mutations of p53 protein and the intensity of it by generating a heatmap using SNAP2, as shown in Figure 7.

### Selection of the PhiKan-083-related analogs

In order to find 100 molecules that are structurally similar to PhiKan-083, the PubChem database was searched (Supplementary Table S1). Following that, the compounds were examined using ADMET analysis. Ten compounds exhibiting specific drug-likeliness characteristics were chosen from the screened compounds, including PhiKan-083 (standard), as shown in Table 1. Using molecular docking studies, the binding affinity of these 10 compounds with TP53 was further evaluated.

**TABLE 1:** ADMET analysis.

S. No.	Compound PubChem CID	Molecular weight g/mol	TPSA Å^2^	GI absorption	Lipinski’s Rule	Ghose rule	Veber rule	Egan rule	Muegge’s rule	ADMET screening
1	16255105	274.79	16.96	High	Yes	Yes	Yes	Yes	Yes	Yes
2	407362	315.43	7.94	High	Yes	Yes	Yes	Yes	Yes	Yes
3	401966	317.39	53.71	High	Yes	Yes	Yes	Yes	Yes	Yes
4	364676	298.81	16.96	High	Yes	Yes	Yes	Yes	Yes	Yes
5	53389	321.46	20.20	High	Yes	Yes	Yes	Yes	Yes	Yes
6	5072	321.46	20.20	High	Yes	Yes	Yes	Yes	Yes	Yes
7	31061	304.43	17.82	High	Yes	Yes	Yes	Yes	Yes	Yes
8	42404	290.40	16.96	High	Yes	Yes	Yes	Yes	Yes	Yes
9	49539	292.42	8.17	High	Yes	Yes	Yes	Yes	Yes	Yes
10	301801	262.35	17.29	High	Yes	Yes	Yes	Yes	Yes	Yes

### Molecular docking analysis

**FIGURE 8:**
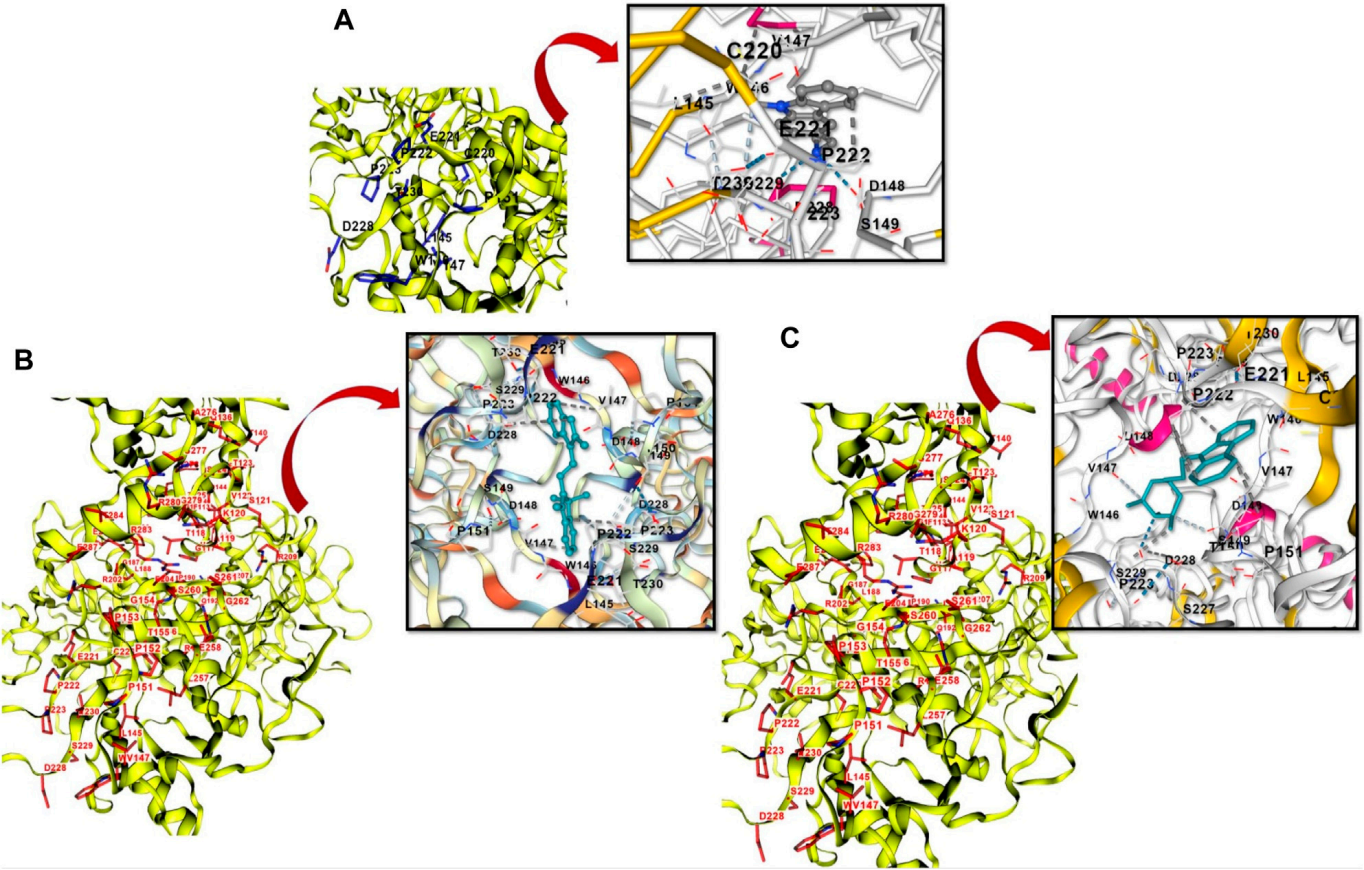
**(A)**: Showing the structure-based cavity binding and docking site between PhiKan-083 and P53. **(B):** Novel PhiKan-083 analog (407362) in binding with P53. **(C):** 53,389, another novel PhiKan-083 analog in binding with P53.

Following ADMET screening, each drug underwent a molecular docking study with the target TP53 (Figure 8). Higher activity of the compound was inferred from the binding affinity of the chosen compounds. According to the study, two ligands with the PubChem IDs 407362 and 53389 displayed higher binding compared to PhiKan-083, the reference molecule (Table 2).

**TABLE 2:** Molecular docking scores of the 10 compounds.

S. No.	IUPAC name	Compound CID	Docking score
**1**	1-(9-ethylcarbazol-3-yl)-N-methylmethanamine; hydrochloride [Standard (PhiKan-083)]	16255105	−7.6
2	1,3,3-Trimethyl-2-[2-(2-methylindol-1-yl)ethenyl]indol-1-ium	407362	−9.6
3	3,6-Bis(4,5-dihydro-1H-imidazol-2-yl)-9-methylcarbazole	401966	−9
4	3-(2,3-dihydro-1H-indol-2-yl)-1,2-dimethylindole; hydrochloride	364676	−9
5	9-[3-[(3R,5S)-3,5-dimethylpiperazin-1-yl]propyl]carbazole	53389	−9
6	9-[3-(3,5-dimethylpiperazin-1-yl)propyl]carbazole	5072	−9
7	6-Methyl-9-[2-(6-methylpyridin-3-yl)ethyl]-1,2,3,4-tetrahydrocarbazole	31061	−9
8	N-methyl-2-(15-methyl-1 azatetracyclo[8.6.1.02,7.014,17]heptadeca-2,4,6,10(17),11,13,15-heptaen-16-yl)ethanamine	42404	−8.9
9	3-Methyl-9-[(1-methylpiperidin-2-yl)methyl]carbazole	49539	−8.9
10	10-Ethyl-1-methyl-3,4-dihydropyrido[3,4-b]carbazole	301801	−8.5

### Result summary

The TIMER 2.0 analysis of the TP53 expression pattern revealed overexpression of TP53 across all TCGA datasets. TP53 levels were further assessed using CPTAC samples from the UALCAN database. After comparing with normal samples, it was also witnessed that the levels of the TP53 protein were significantly elevated in various tumor samples. Through the GEPIA2 database, we further examined the expression pattern of TP53 in several malignancies. These results elucidated that TP53 is highly upregulated in several malignancies, including BLCA, ESCA, BRCA, BRCA basal, BRCA luminal A, BRCA luminal B, and BRCA HER2. Through the Enrichr program, it was witnessed that MF was significantly enriched in comparison to BP and CC through GO analysis. By employing the KM-plotter from GEPIA2, the prognostic importance of TP53 was assessed across all cancer types. As per the study, reduced OS was linked with increased TP53 expression in ACC, BRCA, KIRP, LAML, LGG, LIHC, PRAD, SARC, THCA, and UCS. Elevated TP53 mRNA levels were correlated to worse RFS in LGG, UCS, and BRCA, followed by LAML, SARC, ACC, KIRP, LIHC, PRAD, and THCA, as per the association between TP53 mRNA levels and RFS. These investigations demonstrated the significance of TP53 in cancer prognosis, marking it as a promising target for treating various malignancies. Through the UALCAN database, we analyzed the expression pattern of p53 in breast cancer. Since TNBC is the most aggressive subtype of breast cancer, our study revealed that among all subtypes, P53 was overexpressed in TNBC, and within TNBC, it was most prominent in the BLIS subtype. Since the major problem associated with p53 misfolding is due to its point mutation status, therefore we examined the amino acid sequence of P53 using the GDC (TCGA) database to observe the frequency of the mutational status of P53. It was observed that protein misfolding mainly added to the gain-of-function potential of P53 protein. We further explored the protein secondary structure by exploring the web-based PredictProtein tool and examined the functional effect of point mutations of p53 protein and its intensity by generating a heatmap using SNAP2. In order to analyze the 100 molecules that are structurally similar to PhiKan-083, the PubChem database was searched. Following this, the same compounds were analyzed through ADMET analysis. The top 10 compounds exhibiting specific drug-likeliness characteristics were chosen from the screened compounds, including PhiKan-083 (the standard). Furthermore, by using molecular docking studies, the binding affinity of these 10 compounds with TP53 was evaluated.

## Discussion

In the present investigation, we aimed to explore the expression of TP53 in tumors, its therapeutic consequences, prognostic significance, and the impact of overexpression on overall and relapse-free survival in different types of cancers. We analyzed the TP53 expression profiles across all TCGA tumors using the TIMER 2.0 database. When TP53 was compared to normal samples for the study of differential expression, it was shown that malignancies such BLCA, BRCA, CESC, CHOL, COAD, ESCA, HNSC, KICH, KIRC, and KIRP had elevated TP53 expression. According to this study, TP53 is a major player in a number of carcinomas, making it a worthwhile target. TP53 overexpression was shown to be mostly caused by gene alteration, and TCGA (GDC) was used to evaluate this work. Additional research examining the correlation between TP53’s mRNA expression profile and survival outcome demonstrated a significant association with many malignancies with elevated hazard ratios. The study found a correlation between high TP53 mRNA levels and poor OS in ACC, BRCA, KIRP, LAML, LGG, LIHC, PRAD, SARC, THCA, and UCS. A poorer Relapse-free survival has been associated with higher TP53 mRNA levels in BLCA, CESC, KIRP, KIRC, LIHC, LUAD, and BRCA. Furthermore, we explored the impact of point mutations on p53 protein misfolding using the PredictProtein database. The frequency of genetic alteration played an important role in the gain of tumorigenic functions of P53 protein. Therefore, we examined the potential role of PhiKan-083, a P53 stabilizing agent, and its analogs in breast cancer. We screened 100 analogs to PhiKan-083 and examined them for their drug-likeliness properties. Among them, approximately 10 compounds were selected for their drug-likeliness and further investigated via the molecular docking approach. Then, we attained two novel compound (PubChem IDs 407362 and 53389) analogs to PhiKan-083 that may be further explored for their prospective therapeutic potential by stabilizing P53 misfolding not only for breast cancer but for other carcinomas as well.

## Data Availability

The original contributions presented in the study are included in the article/Supplementary Material; further inquiries can be directed to the corresponding author.

## Abbreviations

ADMET, absorption, distribution, metabolism, excretion, and toxicity; BRCA1/2, breast cancer gene 1/2; BRCA, breast cancer; BLCA, bladder urothelial carcinoma; CESC, cervical and endocervical cancer; CHOL, cholangiocarcinoma; COAD, colon adenocarcinomas; ESCA, esophageal carcinoma; GDC, Genomic Data Commons; GO, Gene ontology; HNSC, head and neck squamous cell cancer; LIHC, liver hepatocellular carcinoma; LUAD, lung adenocarcinomas; LGA, Lamarckian genetic algorithm; MDM2, murine double minute 2; MD, molecular docking; OS, overall survival; PDB, Protein Data Bank; PSA, polar surface area; READ, rectum adenocarcinomas; THYM, thymoma and thymic carcinoma; TCGA, The Cancer Genome Atlas Program; TP53, tumor protein 53; TNBC, triple-negative breast cancer; UCEC, uterine corpus endometrial carcinoma.
