# Corrigendum: Targeting p53 misfolding conundrum by stabilizing agents and their analogues in breast cancer therapy: a comprehensive computational analysis

**DOI:** 10.3389/fphar.2024.1494110

**Published:** 2024-09-30

**Authors:** Burhan Ul Haq, Hina Qayoom, Shazia Sofi, Nusrat Jan, Aisha Shabir, Irshad Ahmad, Fuzail Ahmad, Abdullah Almilaibary, Manzoor A. Mir

**Affiliations:** ^1^ Department of Bioresources, School of Biological Sciences, University of Kashmir, Srinagar, India; ^2^ Department of Medical Rehabilitation Sciences, College of Applied Medical Sciences, King Khalid University, Abha, Saudi Arabia; ^3^ Respiratory Care Department, College of Applied Sciences, Almaarefa University, Diriya, Saudi Arabia; ^4^ Department of Family and Community Medicine, Faculty of Medicine, Al Baha University, Albaha, Saudi Arabia

**Keywords:** breast cancer, mutation, p53 misfolding, aggregation, PhiKan-083, molecular docking

In the published article, there was an error in **Affiliation 3**. Instead of “College of Applied Sciences, Almaarefa University, Diriya, Saudi Arabia,” it should be “Respiratory Care Department, College of Applied Sciences, Almaarefa University, Diriya, Saudi Arabia.”

The authors apologize for this error and state that this does not change the scientific conclusions of the article in any way. The original article has been updated.

